# Diabetes associated pericyte metabolic signatures and pathogenesis of diabetic retinopathy

**DOI:** 10.3389/fendo.2026.1771836

**Published:** 2026-03-06

**Authors:** Casandra Carrillo, Teri L. Belecky-Adams, Nader Sheibani

**Affiliations:** 1 Department of Biology, Indiana University Indianapolis, Indianapolis, IN, United States; 2 Department of Ophthalmology and Visual Sciences, University of Wisconsin School of Medicine and Public Health, Madison, WI, United States

**Keywords:** diabetes, diabetic retinopathy, metabolism, neurovascular unit, pericytes

## Abstract

Pericytes are metabolically active perivascular supporting cells. They are essential for establishing and maintaining the inner blood retinal barrier and retinal neurovascular homeostasis. In diabetic retinopathy, the chronic hyperglycemic environment adversely affects pericyte functions including their glucose flux and metabolism. The early alterations in pericyte metabolic signatures during diabetes make them susceptible as an early damage target. Altered pericyte glucose flux through the polyol pathway, pentose phosphate pathway, hexosamine biosynthesis pathway, protein kinase C pathway, and advanced glycated end products pathway are responsible for mitochondrial dysfunction, inflammation, and oxidative stress. These metabolic changes in pericytes regulate vascular blood flow and tight junctions’ integrity leading to vascular dysfunction and disease progression. This review aims to discuss the current understanding of pericyte metabolic signatures and altered glucose metabolism under hyperglycemic conditions like those present in diabetes. We also aim to highlight the need for further investigation of how pericyte metabolic activities, particularly among pericyte subtypes of the retina and other organs, are coordinated. This knowledge will help to further our understanding of how to preserve the blood tissue barrier and its function, and to prevent not only diabetic retinopathy but also vascular dysfunction in other organs impacted by diabetes.

## 1 Introduction

**Figure 1:**
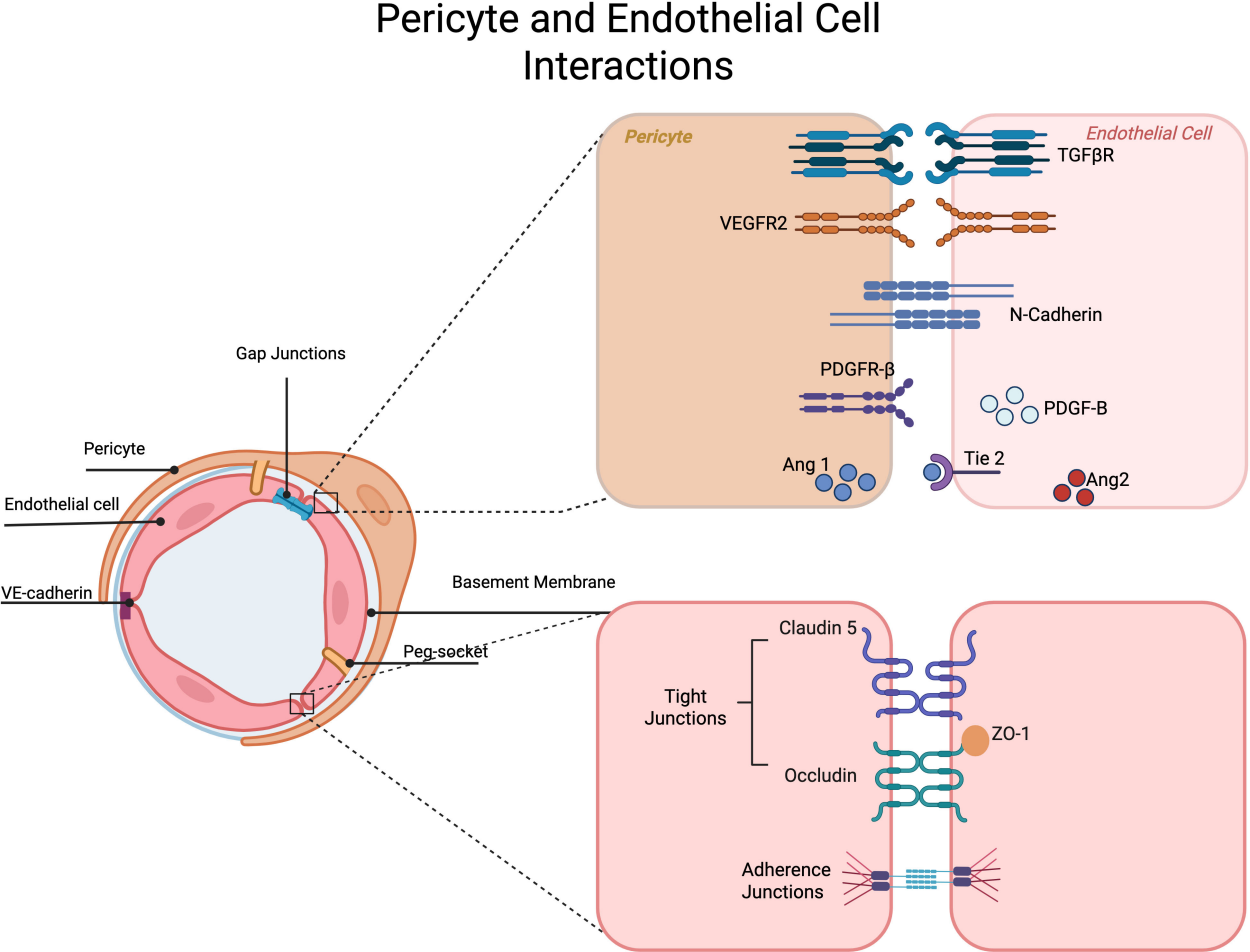
Interactions in Pericytes and Endothelial cells. Various signaling pathways exist between endothelial cells and pericytes. Tight junctions and adherens junctions exist between endothelial cells and pericytes and establish an active barrier between blood components and neuronal tissue.

Pericytes are specialized perivascular mural cells present in the microvasculature of various tissues and organs throughout the body. They originate from the neural crest and/or mesenchymal tissues. The majority of retinal pericytes arise from the neural crest tissue (1–3). Pericytes ensheathe the abluminal surface of endothelial cells and share a basement membrane. Pericytes are unique in that they have long processes that cover the walls of the endothelium, sometimes contacting more than a single endothelial cell and capillary at a time. Given the proximity of pericytes to endothelial cells, they serve essential roles in vascular homeostasis, partly mediated by direct contact and engagement with signaling pathways including vascular endothelial growth factor (VEGF) and VEGF receptors. Pericytes regulate vascular blood flow, sense angiogenic stimuli, and aid in vessel formation and endothelial cell sprouting and survival. Various junctional proteins are integral to maintaining homeostasis and structural stability of the microvasculature throughout the body (4) (**Figure 1**).

The density of pericytes varies among vascular beds of different tissues depending on the organ-specific structure and function. Pericytes are most abundant in the vasculature of the central nervous system, with the retina exhibiting the highest ratio of pericytes to endothelial cells (1:1; in humans) compared to lower ratios in other organs ranging anywhere from 1:10 to 1:100 (5). The high density of pericytes in the neural retina is thought to contribute to the barrier function and structural stability required for retinal function (6). The reason for different endothelial cell and pericyte ratios across tissues remains elusive, but are likely linked to tissue specific functions and metabolic demands (7, 8). Under normal physiological conditions, pericytes exhibit low turnover rates and maintain microvasculature stability over long periods of time. Despite their longevity, pericytes have limited regenerative capability, rendering them vulnerable to pathological stress. In hyperglycemic conditions, pericytes are exposed to high levels of inflammation, oxidative stress, and accumulation of advanced glycation end-products (AGEs) (9). This environment results in pericyte apoptosis or loss, compromising microvasculature stability and promoting microaneurysm formation. Pericyte loss represents one of the earliest events in DR that occurs as a direct result of metabolic dysregulation in the retinal neurovasculature (9).

Retinal neurodegeneration is increasingly recognized to precede vascular abnormalities in diabetic retinopathy (DR) (10). Neuronal stress and glial activation occur before detectable vascular leakage or visible structural damage. Loss of pericyte coverage exacerbates neuronal and endothelial cell damage, contributing to overall barrier breakdown (10). Because pericytes can sense and respond to neuronal stress, their dysfunction can amplify neuronal hypoxia and promote neurodegeneration prior to vascular pathology. Pericyte dysfunction has also been implicated in various diseases including cancer, neurodegenerative diseases, and DR (11). Understanding how pericyte metabolic activity becomes dysregulated under diabetic conditions provides an important link between vascular dysfunction and neurodegeneration, offering broader insight into pericyte metabolic homeostasis.

## 2 The neurovascular unit and blood-retinal barrier

The neurovascular unit (NVU) is a functional complex of highly integrated cells. It is composed of endothelial cells, perivascular supporting cells (smooth muscle cells/pericytes), glial cells, and neurons. The NVU forms not only a structural barrier but also a dynamic signaling unit. Endothelial cells provide a selective barrier, while pericytes stabilize endothelial cells and, consequently, the blood vessels. Neurons and glia generate signals that regulate blood vessel diameter and blood flow. Together, these cells communicate to maintain a highly controlled environment needed for proper neural function by controlling blood flow through neurovascular coupling to match oxygen and glucose demands. The NVU provides the structural and functional framework that supports the integrity of the BRB, a physiological barrier in the retina responsible for regulating nutrients, metabolic waste, and ion flux in and out of the retina (12).

The BRB is a protective structure that actively regulates the exchange of substances between the bloodstream and neural retina. This barrier function and its integrity are dependent on survival of pericytes and endothelial cells, and their proper communication. The newly formed blood vessels attract pericytes to a shared basement membrane by secreting homodimers of platelet-derived growth factor B (PDGFB) by endothelial cells. In turn, pericytes express the receptor for PDGFB (PDGFRβ) (13, 14). The changes in pericyte health with diabetes is thought to be an early event in the retinal vasculature changes that leads to a multitude of alterations including loss of BRB integrity, leaky vasculature, increased inflammation, and potential neuronal loss (13).

In DR increased pericyte loss is one of the earliest signs of the disease (15). Pericyte loss is thought to be a direct result of chronic hyperglycemia and inflammatory responses during diabetes (16). PDGFB is necessary for pericyte migration and survival, which could be inactivated by glycation due to hyperglycemic condition (17, 18). Pericytes may be lost prior to a detectable increase in systemic inflammation. However, recent studies have indicated that pericytes may be lost prior to a detectable increased inflammation in the retina itself (15, 16). The perivascular location of pericytes makes it likely that they are subjected to inflammatory molecules that circulate throughout the bloodstream prior to inflammation in the retina. Pericytes also play an active role in mediating the recruitment of inflammatory cells to sites of inflammation (19).

The presence of advanced glycation end products (AGEs) and oxidative stress lead to the activation of toll-like receptors (TLRs), which are involved in turning on pro-apoptotic factors. Existing research show connections between the chronic inflammation caused by hyperglycemia and progression of DR (20–22). The specific role diabetes-mediated pericyte metabolic changes play in regulation of inflammatory processes in early DR is not fully understood. Given the function of pericytes and their importance in retinal vascular function, inflammation, and maintenance of the BRB, the alterations in their metabolic signatures during diabetes can help elucidate disease states and potential therapies. This review focuses on pericytes and their altered metabolic signatures as an early hallmark of DR. In addition, we will also focus on pericytes within the neurovascular unit, linking pericyte-specific metabolic pathways to early blood retinal barrier breakdown in DR. This review aims to establish a framework for pericyte focused strategies to prevent damage to the NVU that occurs in diseases that lie at the intersection of pericyte metabolism and barrier integrity.

### 2.1 Early changes in DR

DR is a progressive disease, which worsens with longer duration of diabetes. Generally, ocular complications of diabetes are divided into two main stages, the non-proliferative (NPDR) and proliferative DR (PDR) stages based on severity of the retinal neurovasculature damage. Thus, diabetes and its accompanying metabolic signatures affect the retinal NVU; a functional complex comprised of neurons, glial cells, pericytes, and endothelial cells. Collectively, these cellular components maintain the BRB, ensuring homeostasis of retinal neurons by supporting neuronal metabolism and function. DR is marked by retinal neurovascular changes, BRB breakdown, edema, and neovascularization (23).

**Figure 2:**
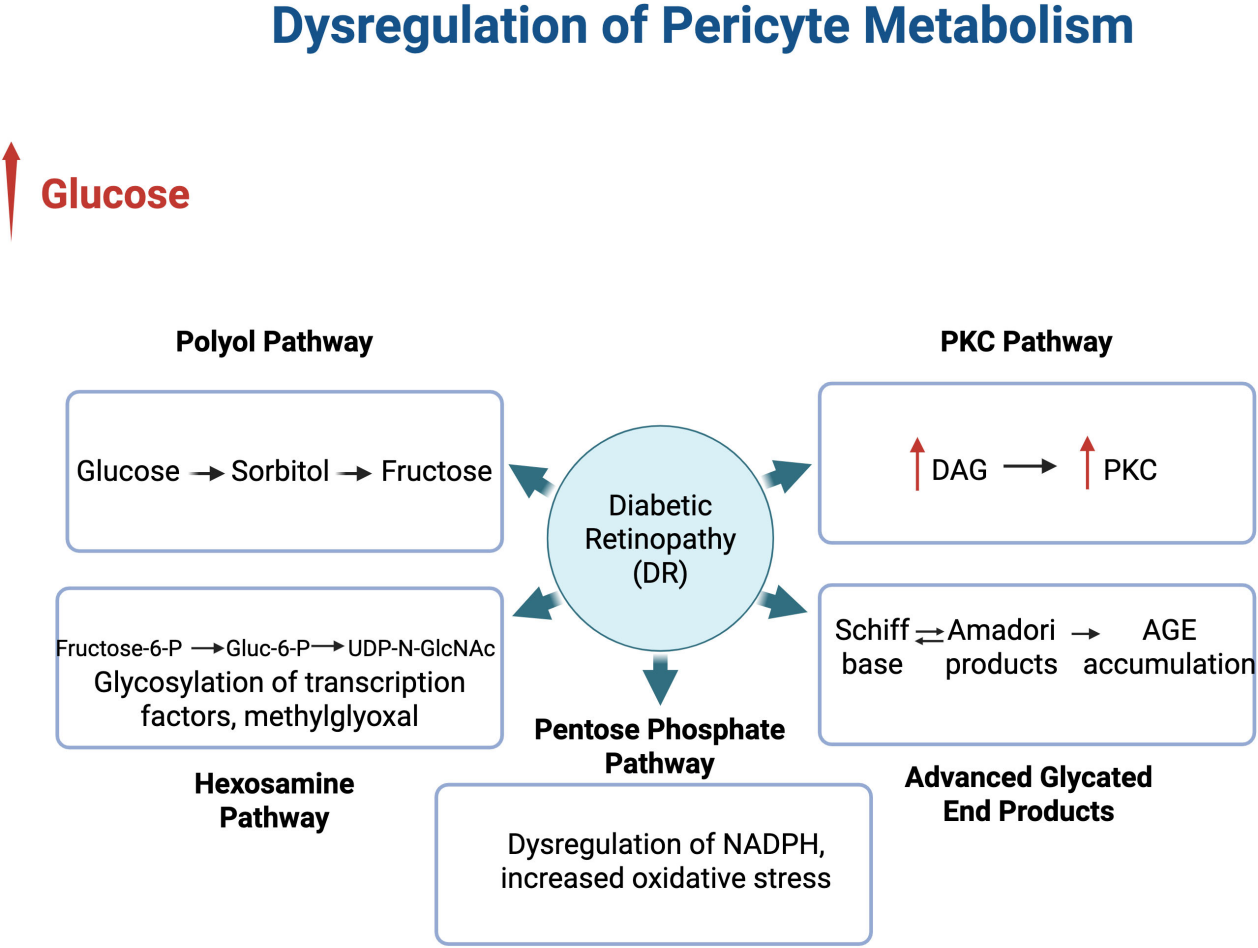
Pericytes and Glia in the NVU. A diagram of pericytes in the NVU and their intracellular connections with other cellular components of the NVU. Physiological conditions are shown above the dotted line, and hyperglycemic conditions are shown below the dotted line. Hyperglycemia affects the NVU by leading to pericyte damage and loss, basement membrane thickening, gliosis, and loss of tight junction proteins.

Morphological vascular changes include pericyte loss, disruption of tight junctions, and increased vascular permeability (24) (**Figure 2**). Weakening of vessel walls leads to saccular dilations that protrude from retinal capillaries, known as microaneurysms. The microvascular abnormalities that reflect ongoing ischemia and indicate progression in disease severity (25). As ischemia worsens, microaneurysm formation and vascular leakage increases, resulting in further vascular permeability and subsequent hemorrhaging. Edema can develop in early non-proliferative DR due to hyperglycemia induced damage and inflammation, disrupting the BRB and occurring even prior to visible hemorrhages are apparent (26).

### 2.2 Late changes in DR

As damaged blood vessels occlude and oxygen levels to the tissue drops, VEGF is released, primarily from Müller glial cells to stimulate the growth of new blood vessels (27). This neovascularization occurs and gives rise to abnormal blood vessels, marking PDR. These fragile new blood vessels create abnormal and leaky vasculature that ultimately interfere with vision. At the end stages, PDR can result in retinal detachment, neovascular glaucoma, shriveling of the eye, and blindness (28). The majority of current research on DR has primarily focused on the later stages of the disease based on clinical diagnosis. However, it is imperative to examine the earliest stages of disease to determine the underlying causes of the disease onset and progression.

The mechanisms involved in the neurovascular dysfunction that drive the development and progression of DR remain largely unknown. While many pathways and factors are involved, dysregulation of retinal pericyte glucose metabolism has been implicated in DR due to chronic hyperglycemia. Hyperglycemia causes increased inflammation, oxidative stress, and impairment of retinal neurovasculature integrity and function. Understanding the specific glucose metabolic signatures of retinal pericytes and maintaining their regulation can provide vital insights for potential therapies for treatment and prevention of DR, as well as other retinal diseases involving pericyte metabolic dysfunctions.

## 3 Glucose metabolism in pericytes

Retina is a highly metabolically active tissue with large oxygen demand satisfied by two circulation systems, namely retinal and choroidal systems. The choroidal vasculature supplies oxygen and nutrients to the outer retina, including the retinal pigment epithelium (RPE) and photoreceptor cells. Choroidal vasculature is separated from the retina by RPE and Bruch’s membrane. RPE cells are responsible for establishing the outer retinal blood barrier. The dysfunctions in this barrier contribute to macular edema and visual impairment. While less studied, recent studies have also shown that diabetes could lead to intra-choroidal neovascularization deep in the choroid and extra choroidal neovascularization within or between Bruch’s membrane and the RPE (29). These extra choroidal new vessels auto infarct, leaving behind collagenous tubes (29). In rodents, retinal vascularization arises from the central retinal artery, which enters at the optic nerve head and branches out across the inner retina through angiogenesis. In humans, the superficial vascular plexus forms by vasculogenesis of preexisting hematopoietic vascular precursor cells, which then extends into the inner retina to form the deep and intermediate capillary plexuses by angiogenesis.

Retinal pericytes are highly influenced by metabolic changes, utilizing different metabolic pathways for their energy needs and function. Glucose metabolism is a large source of energy for pericytes and is necessary for maintaining not only tissue but the entire body homeostasis. Glucose uptake rate in pericytes is much higher than that of endothelial cells (30). Glucose metabolism within pericytes is regulated by various factors that aid in glucose uptake and glycolysis. Glucose taken up from the bloodstream through GLUT1 and GLUT4 transporters, is primarily metabolized by glycolysis. Insulin affects this by increasing the localization of GLUT1 and GLUT4 on the membrane for glucose uptake. The metabolic changes present in diabetes affect all components of the neurovasculature (31). Pericytes normally rely heavily on mitochondrial metabolism, and once glucose makes it inside the cell, it will undergo glycolysis and oxidative phosphorylation. It is known that retinal metabolic changes precede retinal neurovascular cells and tissue damage noted in DR (18, 32). The molecular mechanisms that contribute to the pericyte differences in glucose uptake and its utilization by various intracellular metabolic pathways compared with other cellular components of the neurovasculature will benefit from further investigation.

### 3.1 Pericytes and BRB integrity

Early NPDR is known to be characterized by damage to various components of the inner retinal blood barrier. Tight junctions form the inner blood-retinal barrier are primarily located between endothelial cells, and their expression and function are influenced by signals from pericytes, astrocytes, and Müller glia (12, 31, 33). Metabolic stress affects the endothelial cells, surrounding tissues, and neurons that make up the NVU, and thus the integrity of the inner BRB, which is crucial for oxygen, fluid, and metabolic regulation to maintain retinal homeostasis.

### 3.2 Effects of hyperglycemia

The hyperglycemia that arises in diabetes results in damage and metabolic changes in retinal pericytes (34). Chronic inflammation resulting from hyperglycemia activates oxidative stress and AGE pathways, stimulating the activation of protein kinase C-delta (PKC-δ) (23, 35). Obesity, commonly accompanying diabetes, puts stress on adipose tissue and increases oxidative stress and endoplasmic reticulum (ER) stress. An abundance of adipose tissue macrophages leads to an accumulation of cytokines and chemokines such as tumor necrosis factor-alpha (TNF-α) and interleukin-6 (IL-6) that drive inflammation through interactions with their receptors. These interactions, in turn, activate kinases such as mitogen activated protein kinase (MAPK), JNK, nuclear factor kappa beta (NF-κB), PKC-δ, and ERK signaling. Pericytes are particularly sensitive to PKC-δ mediated apoptosis, leading to increase in pericyte loss. Retinal pericytes are highly sensitive to these signaling pathways because of the relatively low antioxidant capacity and metabolic demands of the retina, which together exacerbate oxidative damage arising from adipose inflammation under hyperglycemic conditions.

Activation of these pro-inflammatory pathways alters insulin signaling, increasing insulin resistance, and contributing to metabolic dysfunction underlying diabetes onset (36, 37). Post-translational modifications and changes to protein levels all drive adipose and metabolic dysregulation (38). Chronic inflammation often persists exacerbating insulin resistance, diabetes, and DR (39). While obesity exacerbates chronic inflammation leading to insulin resistance and type 2 diabetes, type 1 diabetes, an autoimmune disorder, similarly is driven by chronic inflammation. This results in dysregulation of adiposities and glucose metabolism, which further drive hyperglycemia (40). A study using a Streptozotocin (STZ; a drug that mimics type 1 diabetic phenotype) diabetes model demonstrated early retinal inflammation prior to vascular damage (41). This supports inflammation as a primary driver of DR onset in both type 1 and type 2 diabetes (12). Chronic hyperglycemia is responsible for activating various intracellular pathways that specifically target neurovascular cells, particularly pericytes.

## 4 Altered metabolic pathways

Diabetes impacts multiple metabolic pathways in pericyte through osmotic stress and biochemical alterations impairing their function. These changes ultimately drive the development and progression of DR. Metabolic changes due to oxidative stress derived from chronic hyperglycemia impact a number of cellular pathways. These include: 1) Polyol Pathway, 2) Pentose Phosphate Pathway 3), PKC Pathway, 4) Hexosamine Biosynthesis Pathway, and 5) AGE/RAGE. The aberrant engagement of these pathways under high glucose conditions promote mitochondrial dysfunction, DNA damage, inflammation, a reduction in mitophagy, and ultimately pericyte cell death (36, 37).

### 4.1 Polyol pathway

The polyol pathway is a two-step metabolic pathway that serves a key role in microvascular changes under hyperglycemic conditions in the retina. When there is elevated intracellular glucose, this pathway is increasingly activated and responsible for converting glucose into polyols using aldol reductase (AR) and sorbitol dehydrogenase (SDH). Excess glucose that cannot go through normal glycolysis engages in the polyol pathway where AR reduces glucose to sorbitol using NADPH as a cofactor, and SDH oxidizes sorbitol into fructose.

In the first step of this pathway, sorbitol accumulates through increased utilization of the polyol pathway due to excess glucose, which cannot go through normal glycolysis. Chronic hyperglycemia found in diabetes and subsequent DR are known to result in sorbitol buildup (38). This increase in sorbitol poses an issue due to its inability to easily pass through the cell membrane, leading to its intracellular accumulation. AR consumption of NADPH limits the amount of NADPH available to produce glutathione, an antioxidant, leading to increased oxidative stress (39). These observation are consistent with the notion that the global loss of AR prevents diabetes-mediated capillary degeneration and oxidative stress (42).

In the second step of the Polyol pathway, SDH oxidizes sorbitol into fructose using NAD+ as a cofactor. This alters cell metabolism and the ratio of NADH to NAD+ and mimics a hypoxic state contributing to oxidative stress and subsequent damage to the retinal neurovasculature (40). The loss of NAD+ also alters the sirtuins pathway, which is dependent on NAD+ for deacetylation of proteins (41). Conversely, the increase in NADH has inhibitory effects on other metabolic pathways such as glycolysis, fatty acid synthesis, and the Krebs cycle (43–45). By inhibiting these other metabolic pathways, a feedback loop is created where glucose is processed through the polyol pathway, exacerbating this process and retina damage. However, the identity of the cells involved and their cell autonomous contribution to diabetes mediated metabolic changes remain poorly defined.

Oxidative stress damages a range of cell types within the retina during diabetes, including pericytes, endothelial cells, glial cells, and neurons (46). A study on pericytes under high glucose conditions showed high levels of AR. Unfortunately, these studies are limited and done in pericytes in culture, and not animals or tissues *in vivo* (47). The specific role that the polyol pathway plays in pericyte damage and apoptosis is unknown, particularly the specific role of fructose and sorbitol. This leads to the question of how pericytes metabolic signatures are altered with diabetes and how they mediate pericyte damage during diabetes (48).

### 4.2 Pentose phosphate pathway

The Pentose Phosphate Pathway (PPP) is a key branch of glucose metabolism. It contributes to cellular antioxidant defense and biosynthetic processes. The PPP diverts glucose-6-phosphate from glycolysis to generate NADPH through the glucose-6-phosphate dehydrogenase (G6PD). NADPH reducing capacity is vital for antioxidant defense by maintaining reduced glutathione and regenerating thioredoxin, as well as biosynthetic reactions including fatty acid, cholesterol, and nucleotide synthesis (49, 50).

The exposure to chronic hyperglycemia results in elevated oxidative stress in the retina, which puts an increased demand on PPP-derived NADPH. However, excess glucose flux into the polyol pathway consumes NADPH, compromising the cellular antioxidant defense (51). In addition, the rate-limiting enzyme of the PPP, G6PD, is often downregulated or functionally impaired in diabetic tissues, further reducing NADPH production (52, 53). These limitations are particularly detrimental in insulin-independent tissues such as the retina, where unregulated glucose uptake drives oxidative and metabolic stress (54).

The resulting deficiency in reducing equivalents compromises glutathione recycling and other antioxidant mechanisms, making retinal cells more vulnerable to reactive oxygen species (ROS). This imbalance exacerbates endothelial dysfunction, pericyte loss, and microvascular injury, all of which contribute to the pathogenesis of diabetic retinopathy (55, 56). Thus, impaired PPP activity and diminished NADPH generation represent critical mechanisms linking hyperglycemia to oxidative stress and microvascular degeneration in the diabetic retina.

### 4.3 PKC pathway

**Figure 3:**
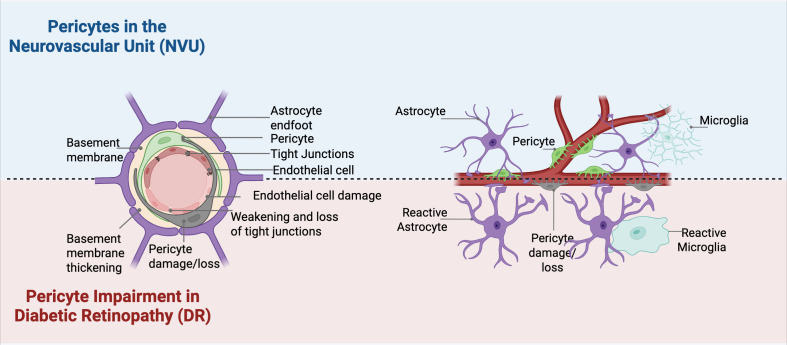
Dysregulation of Metabolic Pathways by Hyperglycemia in Diabetic Retinopathy. The increase in glucose alters metabolic pathways in pericytes that contribute to the development of DR. The polyol pathway leads to an accumulation of sorbitol converted to fructose which then leads to an accumulation of AGEs. The PKC pathway under hyperglycemia is affected by an increase in PKC activation due to an increase in DAG. An increase in hexosamine metabolites contributed to glycosylation of many factors with distinct functions. The Pentose Phosphate Pathway is compromised in its ability to make NADPH, making the retina more susceptible to oxidative stress. These pathways are all impacted by the accumulation of AGEs, a result of Schiff bases and Amadori products.

In addition to the increased activity of the polyol pathway, the protein kinase C (PKC) pathway is also affected (**Figure 3**). The PKC pathway is activated in pericytes under hyperglycemic conditions with important roles in controlling the function of other proteins and can lead to retinal damage. In hyperglycemic conditions, there is an increase in PKC activation through the synthesis of diacylglycerol (57), an endogenous activator of the PKC pathway. Activation of PKC isoforms in endothelial and other retinal cells alters tight junctions between endothelial cells leading to an increase in vascular permeability and leukocyte adhesion (58). This damage to the vasculature leads to abnormal blood flow and thickening of the basement membrane.

The PKC-α isoform is primarily responsible for the damage to the barrier and leakage by phosphorylating tight junction proteins and mediating their dysregulation. PKC-β and PKC-δ isoforms meanwhile are responsible for the phosphorylation of cytoskeletal proteins that lead to changes in tight junction integrity (59–61). Activation of these isoforms also increase inflammatory pathways, which leads to an increase in ICAM-1 expression on endothelial cells. In addition, the activation of these isoforms leads to an increase in leukocyte adhesion and promoting vascular and extracellular matrix remodeling through increased expression of VEGF.

Although PKC isoforms have been implicated in diabetic retinopathy and linked to disrupted nitric oxide signaling and inflammatory responses, effective therapeutic targeting of the PKC pathway remains unresolved due to conflicting results in literature describing involvement of various PKC isoforms. These conflicting results make targeted therapy challenging.

### 4.4 Hexosamine biosynthesis pathway

The hexosamine biosynthesis pathway (HBP) is a metabolic pathway that processes glucose into the production of uridine diphosphate-N-acetylglucosamine (UDP-GlcNAc). Under normal conditions, only a small portion of glucose passes through HBP. When there is excess glucose, more glucose is diverted to HBP, increasing the UDP-GlcNAc production. Glutamine: fructose-6-phosphate aminotransferase (GFAT) is the rate-limiting enzyme in the HBP pathway, using fructose-6-posphate and converting it to glucosamine-6-phospate and glutamate via glutamine. UDP-GlcNAc is involved in intracellular signaling as a substrate for O-GlcNAc transferase, which adds a GlcNAc to serine and threonine residues on proteins, a post-translational modification akin to phosphorylation (62). This post-translational modification is known as *O*-GlcNAcylation and is cycled on and off by two enzymes: *O*-linked β-N-acetylglucosamine transferase (OGT) and *O*-linked β-N-acetlyglucosaminase (OGA) (62).

This post-translational modification plays an essential role in modulation of stability and function of many proteins impacting the cell cycle, and transcriptional and translational processes. These activities are affected by the amount of UDP-GlcNAc present within cells, which is impacted by variations in metabolic activities. Thus, a direct correlation between *O*-GlcNAc and cell nutrient composition exist, allowing *O*-GlcNAc to correlate with many pathologic phenotypes associated with various diseases including DR. DR results from high levels of glucose, and an increase flux through the HBP, leading to higher levels of UDP-GlcNAc, which in turn increase *O*-GlcNAc modification resulting in altered cellular functions, especially in pericytes (63).

An increase in hexosamine metabolites contributes to retinal damage due to the promotion of neuronal death and blocking the insulin signaling pathway (58, 64). This pathway also increases oxidative stress and disrupts the regulation of retinal inflammatory responses. These metabolic changes affect pericyte survival and functionality, leading to their dysfunction and dropout. Given the key role of pericytes in the retinal vasculature, their loss results in the loss of regulation in vascular tone and perfusion pressure in capillaries. These metabolic and functional alterations because of hyperglycemia contribute to vascular instability. Hyperglycemia also leads to mitochondrial fragmentation and dysfunction of retinal pericytes. This leads to a decrease in oxygenation and ultimate pericyte loss. The selective increased *O*-GlcNAc modification under high glucose conditions affect retinal pericytes more than other retinal cell types, perhaps due to increased glucose intake by pericytes, suggesting a contribution to the early loss of retinal pericytes during diabetes and development and progression of DR (65).

There remains a significant gap in our understanding of the regulation of this pathway when it comes to pericyte glucose metabolism. While the literature suggests that pericytes move glucose through the HBP at a higher rate than other cells like endothelial cells and astrocytes under high glucose conditions, studies exploring the reasoning and mechanisms behind this remain elusive (62, 65). As mentioned earlier, metabolic studies on pericytes have been mainly performed in cell culture, and have not explored the various metabolic pathways, including HBP *in vivo*. While HBP has been implicated in disease states, how this specifically applies to pericytes, their fate, and their role in vessel stability, particularly during diabetes deserves further exploration (62, 66).

### 4.5 AGEs formation and RAGE signaling

Under hyperglycemic conditions, like those present in diabetes, glucose and glucose metabolites undergo various non-enzymatic reactions that lead to the accumulation of toxic byproducts, including advanced glycation end-products (AGEs). As mentioned, under hyperglycemia, excess glucose is partly processed into the polyol pathway, which temporarily reduces intracellular glucose levels. However, sorbitol builds up within cells, resulting in cell swelling, stress, and metabolic dysfunction (42, 67).

Excess sorbitol in cells is converted into fructose by sorbitol dehydrogenase. Fructose is much more reactive to the formation of AGEs than glucose. Fructose is further broken down by phosphorylation and turned into fructose-3-phosphate, which spontaneously breaks down into 3-deoxyglucosone, a precursor of AGEs. The enzyme responsible for the conversion of glucose to sorbitol needs NADPH. This greatly reduces the amount of NADPH that is available for glutathione reductase, an enzyme critical for antioxidant defense. These collectively lead to an increase in oxidative stress, further increasing AGEs formation.

The accumulation of AGEs is a key contributor to the progression of DR and its subsequent vascular complications (68–71). This accumulation of AGE is directly detrimental to pericytes, which are especially sensitive to AGEs. An AGE rich environment leads to pericyte dysfunction and cell death. The loss of pericytes, an early hallmark of DR, leads to vascular dysfunction and permeability as well as ischemia.

The Receptor for Advanced Glycation End Products (72) medicates AGEs responses and is critical in amplifying many of its detrimental effects. RAGE is a receptor that is highly expressed in the retina, immune cells, endothelial cells, pericytes, and other neurons in the retina. When AGEs bind RAGE, engagement of intracellular signaling pathways leads to a proinflammatory state. One of the main pathways activated by AGE-RAGE binding is NF-κB, a transcription factor with key roles in inflammation. When AGE-RAGE binding occurs, a signaling cascade activates proteins and kinases which eventually converge on IκB, whose phosphorylation results in degradations allowing translocation of NF-κB from the cytoplasm to the nucleus promoting transcription of pro-inflammatory genes (73–75). Upregulation of transcription factors for chemokines, cytokines, and adhesion molecules all exacerbate inflammation and vascular dysfunction. NF-κB also promotes BRB breakdown, vascular dysfunction, and promotes DR progression (12, 76, 77).

## 5 Oxidative stress, inflammation, and cell death

Increased ROS and oxidative stress damage mitochondrial DNA, disrupt cellular metabolism, and exacerbate insulin dysregulation (78, 79). Mitochondria undergo structural changes where they become swollen (66), impacting membrane potential and mitochondrial fragmentation in retinal vascular cells (67). Along with this damage, there is a reduction in mitophagy, the process of removing damaged mitochondria, leading to accumulation of damaged mitochondria further contributing to systemic inflammation. In addition, mitophagy is also altered in pericytes, contributing to their death in response to hyperglycemic conditions. Retinal cultures show an increase in mitophagy in response to AGEs treatment supporting the disruption of mitophagy in response to hyperglycemia and oxidative stress.

Several markers of autophagy, microtubule-associated protein 1A/1B-light chain 3B (LC3B), Beclin-1, and Atg5 are all increased in retinal pericytes of diabetic mice (80–82). In addition, autophagosomes and autolysosomes are found in retinal pericytes and vascular smooth muscle cells (83). Autophagy is known to modulate pericyte migration and contributes to pericyte loss and autophagic activity accompanies the presence of pericyte ghosts found in DR (83). Due to the ability of ROS and AGEs to activate autophagy, the inhibition of autophagy could reduce AGE-induced migration of pericytes (84). Addressing autophagy and its modulation can lead to the preservation of pericyte and overall integrity in DR.

The inflammatory cytokines, IL-6, TNF-α, and IL-1β are primarily secreted by Müller glia, retinal microglia, and endothelial cells in response to hyperglycemia. Once secreted, these cytokines bind TNRF1 receptors on pericytes, altering their ability to proliferate and migrate. At the same time, ICAM-1 and VCAM-1 are upregulated in endothelial cells, increasing leukocyte adhesion and migration (85). These cytokines converge downstream of the five major pathways affected by diabetic metabolic dysregulation, leading to ROS generation and NF-κB activation (86, 87).

Intracellular hyperglycemia elevates ROS production inducing DNA damage. This damage inactivates GAPDH, diverting glucose metabolism into parallel pathways. Flux through the polyol pathway increases, which in turn enhances AGE formation, activates PKC, and elevates the hexosamine pathway activity. These events converge on NF-κB activation and subsequent inflammation (88).

IL-1β specifically induces pericyte apoptosis through the NF-κB pathway under hyperglycemic conditions while also degrading endothelial tight junction proteins and increasing vascular permeability (89–91). In addition to these cytokines, IFNγ is a key mediator that disrupts PDGFRβ signaling, promoting its degradation and subsequently elevating complement C3 (CC3) and PKC- δ, which directly contributes to pericyte dropout and BRB damage. Work in our laboratory has shown that chronic exposure to IFNɣ reduces PDGFRβ signaling and increases pro-apoptotic factors including CC3 and PKC-δ, leading to pericyte death. By impairing PDGFRβ signaling and inhibiting pericyte migration, IFNγ is responsible for BRB dysfunction (72, 92). Together, these cytokines create profound metabolic and signaling imbalances that drive pericyte loss (16).

## 6 T1D vs. T2D: subtype heterogeneity

The long-term effects of altered glucose metabolism on pericyte function and integrity in DR are not fully understood, particularly the differences between type 1 diabetes (T1D) and type 2 diabetes (T2D). Both T1D and T2D result in pericyte metabolic dysfunction and overall cell loss. However, the way each disease leads to increased glucose levels differ. In T1D, pericytes are exposed to high glucose levels due to insulin deficiency, while in T2D, pericytes are more likely to be exposed to fluctuating glucose levels with a more gradual progression to hyperglycemia. In T1D, due to the absence of insulin, pericyte dysfunction occurs early, sometimes even before a clinical diagnosis (93, 94).

Although T1D and T2D both result in the downregulation of GLUT transporters, an increase in oxidative stress, early pericyte loss, and progression of DR there may be differing underlying mechanisms, potentially leading to different susceptibilities and progression on neurovascular damage. However, there is a lack of knowledge regarding pericyte glucose metabolism and DR resulting from T1D and T2D. The onset and duration between T1D and T2D make it difficult to compare the two. Pericyte ability to differentiate to myofibroblasts is not prominently seen in T2D but much more commonly seen in T1D (93, 95). While both types of diabetes affect glucose transporters, GLUT1 is downregulated in both, GLUT2 is more commonly seen as being affected in T2D (96, 97). However, using murine models of T1D and T2D we found that both show a similar progression of pericyte loss (16). It is important to note that in T2D, DR takes longer to be diagnosed, leading to many years of undetected hyperglycemia compared to T1D.

To further complicate how pericyte glucose metabolism is impacted during diabetes, there is also evidence that there are different populations of pericytes in the retina, which are targeted by diabetes (2, 15). Two main subsects of capillary pericytes are essential to BRB maintenance. These include perivascular pericytes which are thought to have a phagocytic function and aid in debris clearance (98). During DR, pericyte loss is apparent across the entire retina but capillary pericytes appear to be most affected (15, 99). In the early stages of DR, the pericyte to endothelial cell ratio decreases from 1:1 to 1:4 (15).

Defining the subtypes of pericytes has been challenging due to the changes that pericytes undergo based on their environment as well as pericyte isolation (100, 101). Attempts have been made to classify pericytes using immunofluorescence to determine their location and differentiation (100). However, pericytes and other cells express similar biomarkers, making distinguishing pericytes from other cell types much more difficult by immunofluorescent markers. Studies most commonly must perform immunofluorescence with multiple pericyte markers to attempt to differentiate subtypes. Capillary and perivascular pericytes are some subtypes that have been differentiated based on marker combinations. Capillary pericytes are positive for neural glial antigen 2 (NG2) marker and negative for Desmin and alpha smooth muscle actin (α-SMA). Meanwhile, perivascular pericytes are positive for all three markers: NG2, Desmin, and α-SMA (102).

## 7 Therapeutic implications

Given major pathways in glucose metabolism drive hyperglycemic dysregulation, inhibitors of these pathways show some potential as they may be an avenue to reduce osmotic stress and ROS production but face selectivity challenges. Aldose reductase inhibitors (ALRi) block polyol pathway activation, shown in preclinical models, although ALR2 specificity is difficult to achieve with >50% homology with ALR1 which is essential to detox the body (103). PKC-ß inhibitor ruboxistaurin was shown to preserve tight junction integrity in clinical trials, and while it reduced vision loss, did not prevent DR progression (104).

Targeting inflammation is an attractive approach, in animal studies, JACK-STAT inhibitors halt feedback loop (105). Antioxidant treatments target impairment of the PPP pathway, mitigating mitochondrial damage due to oxidative stress (106). Enhancing DJ-1/nuclear factor erythroid 2-related factor 2 (Nrf2) antioxidant axis represents an additional approach. DJ-1 overexpression activated Nrf2 signaling, upregulating antioxidant enzymes such as MnSOD and catalase, reducing ROS production, and shifting the Bcl-2/BAX ratio toward pericyte survival (107). Isoform specificity and pathway crosstalk make it essential to optimize optimal downstream targets.

Modulation of the nicotinamide adenine dinucleotide (NAD+) sirtuins (SIRT1) pathway has also emerged as a promising pericyte-directed strategy. SIRT1 expression is reduced in diabetic retinas, and intravitreal delivery of AAV2-SIRT1 in diabetic mice increased retinal SIRT1 levels, reduced CC3 activation, decreased hypoxia inducible factor 1α (HIF1α) expression, and reversed functional damage (108). Additionally, inhibition of exchange protein directly activated by cAMP1 (Epac1) signaling shows therapeutic potential: Epac1 is upregulated in diabetic retinal pericytes and promotes mitochondrial fission via DJ-1 phosphorylation, leading to ROS production and CC3 activation. Genetic deletion or pharmacological inhibition of Epac1 blocked high glucose-induced mitochondrial dysfunction and protected against pericyte loss (109).

Recent single-cell transcriptomic studies have identified pituitary tumor-transforming gene 1 (PTTG1) as a novel metabolic target. A pericyte subcluster with elevated PTTG1 expression correlates with diabetic microvascular dysfunction, and PTTG1 knockdown reprogrammed pericyte energy metabolism by modulating glycolysis pathway genes, reduced oxidative stress, and improved retinal vascular integrity in diabetic mice (110).

New studies on pericyte-specific therapies show promise as they focus on pericyte subtype heterogeneity (111). Through this approach, targeting NG2+ pericytes with antagonists to RAGE or PDGF-BB could protect the pericytes which are targets in early DR (112). Leaning into metabolic intervention prioritizes pericyte preservation can change the management and treatment of DR. Modulation of pericyte glucose metabolism offers a promising new target in early DR intervention.

## 8 Conclusions and future directions

Pericytes are critical for development and maintaining BRB integrity. In diseases like DR, pericytes are altered due to metabolic and inflammatory changes that contribute to disease onset and progression. This review highlighted some of the pericyte metabolic pathways and how they are impacted by chronic hyperglycemia, leading to oxidative stress, AGE accumulation, inflammation, and mitochondrial dysfunction. These changes negatively affect pericyte survival and function, promoting destabilization of retinal vasculature and progression of DR (**Figure 3**). The gaps that currently exist in pericyte metabolic alterations during diabetes also leave major limitations in our current understanding of pericyte subtype populations within the retina. Recent studies on pericytes have suggested that pericytes are heterogenous, not a homogenous cell type like was previously thought (113). This opens the possibility that pericytes can vary in their function and critically, in their metabolic and inflammatory responses under hyperglycemic conditions like those of diabetes (114, 115). This is a critical gap when studying DR and pericyte dysfunction and loss as an early hallmark of the disease especially if various pericyte subtypes exist within the BRB.

Future studies should focus on further investigating the pericyte glucose metabolic signatures changes during diabetes, particularly in the characterization of retinal pericyte subtypes and their unique metabolic needs. The different subtypes of pericytes may vary in their tolerance of hyperglycemic conditions, with some being more tolerant of hyperglycemic stress while others are more susceptible. The treatment of DR by replacing lost pericytes using differentiated stem cells and reestablishing the BRB has been proposed. However, the replacement of these pericytes stands to be a temporary solution to a systemic problem due to persistently aberrant glucose metabolism and vascular inflammation within the diabetic milieu. Various types of stem cells have been tested in animal models of DR, but due to the complexity of DR, identifying the best candidate for restoring pericytes integrity and function remains a challenge (116). Assuming uniformity in pericytes can hinder stem cell therapeutic approaches to treating DR and preventing progression of the disease. Further investigating pericyte heterogeneity can highlight how these subtypes may interact with other components of the NVU in establishing the BRB allowing the development of more effective treatment modalities.

Additionally, major questions remain about the specific biochemical pathways explored in this review. While polyol pathway activation and accumulation of fructose and sorbitol are implicated in hyperglycemic-induced damage, the precise metabolites that govern pericyte dysfunction and survival remain largely unknown. The hexosamine biosynthetic pathway is critical for linking altered glucose metabolism to glycosylation and ECM production, yet its downstream effects on pericytes and endothelial cells remain unclear. Many studies have been conducted *in vitro*, using isolated cell lines under controlled conditions, leaving important gaps in our understanding of pericyte glucose metabolism and limiting our ability to draw definitive conclusions. These remaining gaps in our understanding of pericyte glucose metabolism limit our ability to draw definitive conclusions. Addressing these limitations will require carefully designed studies that specifically trace pericyte metabolism and rigorously characterize pericytes and endothelial cells during targeted manipulation of these pathways. *In vivo* approaches to studying pericyte metabolic demands will be particularly valuable for linking metabolic alterations to structural and vascular changes in chronic hyperglycemic models.

Together, the findings summarized in this review aim to highlight that pericyte metabolic integrity and mitochondrial homeostasis are crucial to retinal stability and represent an essential link between metabolic dysfunction and vascular pathology in DR. Deeper insight into pericyte metabolism *in vivo* and pericyte heterogeneity may be key to developing DR therapies that prevent disease progression and restore vascular health.
